# Effects of Hydrogen Peroxide Stress on the Nucleolar Redox Environment and Pre-rRNA Maturation

**DOI:** 10.3389/fmolb.2021.678488

**Published:** 2021-04-26

**Authors:** Russell T. Sapio, Chelsea J. Burns, Dimitri G. Pestov

**Affiliations:** ^1^Graduate School of Biomedical Sciences, Rowan University School of Osteopathic Medicine, Stratford, NJ, United States; ^2^Department of Cell Biology and Neuroscience, Rowan University School of Osteopathic Medicine, Stratford, NJ, United States

**Keywords:** oxidative stress, H_2_O_2_, nucleolus, ribosome biogenesis, rRNA, RNA processing, roGFP, catalase

## Abstract

Identifying biologically relevant molecular targets of oxidative stress may provide new insights into disease mechanisms and accelerate development of novel biomarkers. Ribosome biogenesis is a fundamental prerequisite for cellular protein synthesis, but how oxidative stress affects ribosome biogenesis has not been clearly established. To monitor and control the redox environment of ribosome biogenesis, we targeted a redox-sensitive roGFP reporter and catalase, a highly efficient H_2_O_2_ scavenger, to the nucleolus, the primary site for transcription and processing of rRNA in eukaryotic cells. Imaging of mouse 3T3 cells exposed to non-cytotoxic H_2_O_2_ concentrations revealed increased oxidation of the nucleolar environment accompanied by a detectable increase in the oxidative damage marker 8-oxo-G in nucleolar RNA. Analysis of pre-rRNA processing showed a complex pattern of alterations in pre-rRNA maturation in the presence of H_2_O_2_, including inhibition of the transcription and processing of the primary 47S transcript, accumulation of 18S precursors, and inefficient 3′-end processing of 5.8S rRNA. This work introduces new tools for studies of the redox biology of the mammalian nucleolus and identifies pre-rRNA maturation steps sensitive to H_2_O_2_ stress.

## Introduction

Throughout their lifespan, cells must defend themselves against reactive chemical species capable of modifying and damaging cellular components. Reactive oxygen species (ROS) are generated in a wide range of biochemical processes and can play both physiologically beneficial and harmful roles in cells (Winterbourn, 2008; Liochev, 2013). Excessive levels of ROS, if not properly controlled, can lead to damage in cellular macromolecules, contributing to the loss of homeostasis in a large variety of diseases and aging (Halliwell, 2006; Sohal and Orr, 2012). Like other types of biological molecules, RNA is susceptible to oxidative damage (Küpfer and Leumann, 2014; Nunomura et al., 2017; Xu et al., 2020), but many aspects of ROS-induced effects on RNA remain incompletely understood.

Ribosomes are complex ribonucleoprotein machines that translate genetic information into proteins. Molecular changes driven by ROS have been observed in both ribosomal RNA (rRNA) and ribosomal proteins (Caldwell et al., 1978; Mirzaei and Regnier, 2006; Liu et al., 2012; Shedlovskiy et al., 2017; Topf et al., 2018), found to increase in human pathologies such as Alzheimer’s disease (Honda et al., 2005; Ding et al., 2006), and shown to affect ribosome functionality (Willi et al., 2018). In our studies, we set out to investigate how oxidative stress affects ribosome biogenesis, an essential process by which cells build and maintain their translation machinery. Under favorable conditions, cells allocate a substantial share of their resources to the synthesis of new ribosomes (Warner, 1999; Dai and Zhu, 2020). In eukaryotic cells, transcription of the large primary rRNA precursor by RNA Pol I and most steps in processing of the pre-rRNA into mature rRNAs occur in the nucleolus, a nuclear compartment hosting the complex molecular machinery of ribosome assembly (Henras et al., 2015; Baßler and Hurt, 2019). Impairment of ribosome biogenesis can trigger signaling responses with major biological consequences, such as selective losses of cells within the hematopoietic system and certain other cell lineages (Danilova and Gazda, 2015; Farley and Baserga, 2016), and may also result in detrimental changes in the proteome (Mills and Green, 2017).

Aside from genetic causes, disruptions in ribosome biogenesis have been studied mainly in the context of small-molecule inhibitors of pre-rRNA transcription and maturation (Burger et al., 2010; Awad et al., 2019). By contrast, a systematic analysis of effects of oxidative stress on ribosome biogenesis and pre-rRNA metabolism has been lacking. One of the current barriers to studying oxidant-driven effects on pre-rRNA is the paucity of tools to selectively regulate and monitor the redox environment of ribosome assembly. Here, we developed a reporter based on a redox-sensitive green fluorescent protein (roGFP) to investigate the impact of ROS on mammalian ribosome biogenesis. We challenged mouse 3T3 cells with low, sublethal doses of hydrogen peroxide (H_2_O_2_) and examined its effects on pre-rRNA while deploying a conditionally expressed, nucleolar-targeted catalase to specifically modulate H_2_O_2_ levels around the sites of pre-rRNA generation and processing.

## Materials and Methods

### Antibodies

The antibodies and their dilutions were as follows: anti-DNA/RNA Damage antibody clone 15A3, 1:500 (Sigma, Cat# SAC5200010); anti-Nucleophosmin 1 (NPM1) antibody clone FC-61991, 1:2,000 (Invitrogen, Cat# 32-5200); anti-β-actin clone C4, 1:10,000 (MP Biomedicals Cat# 0869100); anti-FLAG tag polyclonal antibody for immunofluorescence, 1:1,000 (Invitrogen, Cat# PA1-984B); anti-FLAG tag antibody for immunoblotting clone M2, 1:2,000 (Sigma, Cat# F1804); anti-mouse IgG-HRP conjugate (Millipore, Cat# 06-371); anti-rabbit IgG Alexa Fluor 488 conjugate (Invitrogen, Cat# A-11034); anti-mouse IgG Alexa Fluor 647 conjugate (Invitrogen, Cat# A-21235).

### Plasmid Constructs

The Nucleolar ROS reporter–catalase construct (pNoROS-CAT) was generated using the Sleeping Beauty-GFP-Puro (pSBtet-GP) expression cassette as the backbone (a gift from Eric Kowarz, Addgene plasmid #60495) (Kowarz et al., 2015). The puromycin selectable marker was swapped for a blasticidin selectable marker through Gibson assembly. Redox sensitive green fluorescent protein 2 (referred to herein as roGFP) was cloned from pGDP-roGFP (a gift from Paul Schumacker, Addgene plasmid #49436) (Waypa et al., 2010), fused with an N-terminal nucleolar localization sequence (NoLS) and cloned downstream of the RPBSA constitutive promoter to replace wild-type GFP in pSBtet-GP using Gibson assembly. Mouse catalase coding sequence was cloned from the NIH 3T3 cell RNA by using reverse transcription-PCR, fused with an N-terminal NoLS and C-terminal FLAG tag, and cloned downstream of the Tet-On promoter in place of Luc-Firefly in pSBtet-GP using Gibson assembly. The pNoROS-CAT plasmid will be made available through Addgene.

### Cell Culture and Treatment Conditions

Except where specified otherwise, NIH 3T3 cells used in this study were maintained at 37°C, 10% CO_2_ in DMEM (Corning Cat# 10-017-CV) supplemented with 10% calf serum (HyClone Cat# SH30072) and 1% penicillin-streptomycin (Corning Cat# 30-002-CI). Cells were regularly tested for the absence of mycoplasma. Cells were cotransfected with pNoROS-CAT and the modified Sleeping Beauty transposase vector pCMV(CAT)T7-SB100 (a gift from Zsuzsanna Izsvak, Addgene plasmid #34879) (Mátés et al., 2009) using PolyFect (Qiagen Cat# 301105) following the manufacturer’s instructions. The 3T3-pNoROS-CAT stably transfected pools were selected with 10 μg/ml of blasticidin S (Gibco Cat# R21001) for 7 days. Hydrogen peroxide used for cell treatments was from Macron (Cat# 5240), doxycycline (Dox) was from Frontier Scientific (Cat# D10056).

### Flow Cytometry

pNoROS-CAT-transfected and untransfected cells were seeded into a six-well plate (1.5 × 10^5^ cells per well). The following day, attached cells were washed once with ice-cold PBS, scraped off the plate into 1 ml of PBS, and kept on ice prior to analysis. roGFP expression was assayed using an Accuri C6 Flow Cytometer with excitation at 488 nm and a 533/30 nm emission filter.

### Ratiometric Analysis of NoLS-roGFP

A total of 8.0 × 10^4^ 3T3-pNoROS-CAT cells were seeded into a 35 mm glass-bottom dish (Matsunami Cat# D113OH) using the low autofluorescence FluoroBrite DMEM (Gibco Cat# A18967-01) supplemented with 10% calf serum, 1% penicillin-streptomycin, and 4 mM GlutaMax (Gibco Cat# 35050-061). To induce NoLS-CAT expression, 1 μg/ml doxycycline was added to the medium 24 h prior to assays. For live-cell imaging, the dish was placed into a Tokai Hit INUG2A-TIZ stage top incubator set to the manufacturer’s recommendations (5% CO_2_) 15 min prior to imaging to allow the cells time to equilibrate to the incubator’s environment. After this incubation, samples were imaged using a Nikon Eclipse Ti confocal microscope with a C2 Si scanning head. 7 × 0.75 μm image stacks were collected, first by exciting the samples with a 404 nm laser and then a 488 nm laser. A 515 nm emission filter was used for each image acquisition. After taking the baseline no-treatment (NT) images, samples were treated with 200 μM H_2_O_2_ and imaged 30 min and 2 h later. A composite image using the sum intensity (Composite Sum-Intensity Image, CSII) was generated in ImageJ for each z-stack excited at 404 and 488 nm. Regions of Interest (ROIs) around the nucleoli were generated by creating a binary mask of a composite of the 404 nm and 488 nm CSIIs and adjusting the local threshold using the Bernsen method with the radius set to 15 pixels. ROI noise that fell outside of the nucleus was manually deleted and then the mask was applied to the 404 nm CSII and 488 nm CSII to measure the signal intensity. To determine the relative oxidative state of the nucleoli, signal intensities of each ROI at 404 nm were divided by those at 488 nm.

### Immunofluorescence to Detect 8-oxo-G in Nucleolar RNA

Glass coverslips were washed in 70% ethanol, flamed, dried, and placed into a six-well cell culture plate. After allowing the coverslips to cool, 7.5 × 10^4^ 3T3-NoROS-CAT cells were seeded per well. To induce NoLS-CAT expression, cell culture medium was supplemented with 1 μg/ml Dox for 24 h prior to the experiment. Oxidative stress was induced with H_2_O_2_ added to medium to 200 μM for 30 min to 2 h. The following steps were performed at room temperature. The coverslips were washed once with PBS and fixed for 10 min in 4% paraformaldehyde-PBS. Following fixation, coverslips were washed three times in PBS and permeabilized for 5 min in PBS/0.2% Triton X-100. After permeabilization, the coverslips were washed three times in PBS and then 1–2 drops of Image-iT FX signal enhancer (Invitrogen Cat# I36933) were added to each coverslip followed by incubation for 30 min. The inclusion of the Image-iT FX step was critical for reducing background staining in the protocol. Coverslips were then washed three more times in PBS and blocked for 1 h in PBST (PBS with 0.1% Tween 20) supplemented with 5% goat serum (MP Biomedicals Cat# 2939149). Samples were probed with mouse 8-oxo-G antibody in 1% goat serum-PBST for 1 h, followed by three 10-min washes in PBST, and incubated with anti-mouse Alexa Fluor 647 in 1% goat serum-PBST for 1 h in a dark chamber. Every subsequent step was done under a weak diffused light. After the secondary antibodies, coverslips were washed three times, 10 min each, and stained with 2 μg/ml Hoechst 33342 (Sigma Cat# B2261) in PBST for 5 min, washed again for 5 min in PBST, and stained with 0.5 μM pyronin Y (Sigma, Cat# 83200) in PBST for 30 s. For cell imaging, coverslips were quickly rinsed in water and mounted with ProLong Gold (Invitrogen Cat# P36930).

Fixed cells were imaged the next day using a Keyence microscope (BZ-X710, 100 × objective). Five 0.5 μm images were taken per z-stack. The data were analyzed using ImageJ. The z-stacks for Hoechst, pyronin Y and 8-oxo-G staining were used to generate a separate CSII for each signal. ROIs around nucleoli were created by adjusting the local threshold of the CSII of pyronin Y using the Bernsen auto local threshold method setting the radius to 15 pixels. The ROI noise that fell outside of the nucleus was manually deleted and the remaining nucleolar ROI masks were applied to the 8-oxo-G CSII. The 8-oxo-G intensities were divided by the area (pixels) of each ROI.

### DNase and RNase Treatment

Cells were plated on 18 × 18 mm glass coverslips coated with poly-L-lysine to increase cell adherence during DNase/RNase treatments. For coating, coverslips were washed in 70% ethanol, sterilized by flaming, dried and treated with 0.01% poly-L-lysine in PBS by rocking slowly for 10 min. Next, the coverslips were briefly rinsed in PBS and dried in a 37°C incubator for 120 min. A total of 7.5 × 10^4^ cells were seeded per coverslip into each well. The following day, attached cells were washed with PBS and permeabilized in 10 mM PIPES-NaOH pH 7.0, 100 mM NaCl, 300 mM sucrose, 3 mM MgCl_2_, 0.02% digitonin for 5 min. The coverslips were next washed with PBS and treated with either 250 U/ml of DNase I (Worthington, DPRF grade) or an RNase A/T1 mix (200 μg/ml RNase A, 500 U/ml RNase T1, Thermo Scientific Cat# EN0551) for 20 min at room temperature in 40 mM Tris–HCl pH 7.4, 147 mM NaCl, 2.7 mM KCl, 10 mM CaCl_2_, 6 mM MgCl_2_. After the DNase/RNase treatment, the coverslips were rinsed with PBS and the cells were fixed with glyoxal solution (PBS, 3.15% glyoxal, 19.87% EtOH, 0.76% acetic acid, pH 4.4) for 10 min (Richter et al., 2018), washed 3 times with PBS and then further stained and analyzed for immunofluorescence as described for 8-oxo-G detection.

### RNA Analysis

Total RNA was isolated from cells using RNAzol RT (Molecular Research Center), dissolved in formamide and run on agarose gels, blotted and hybridized as previously described (Wang and Pestov, 2016). Small pre-rRNA intermediates were separated using polyacrylamide gels (Wang et al., 2015). Quantitative analysis of pre-rRNA ratios was performed according to (Wang et al., 2014).

### Software and Data Analysis

We used GraphPad Prism 8.4.3 for statistical analysis, FlowJo v.10 to analyze flow cytometry data, Nikon software NIS-Elements AR 4.30.02 to analyze roGFP ratios in live cell images, and the Fiji distribution of ImageJ (Schindelin et al., 2012) for all other microscopy analysis. Images for publication were prepared using Canvas X (ACD Systems, Inc.).

## Results

### Design and Development of a Nucleolar roGFP Reporter

**FIGURE 1:**
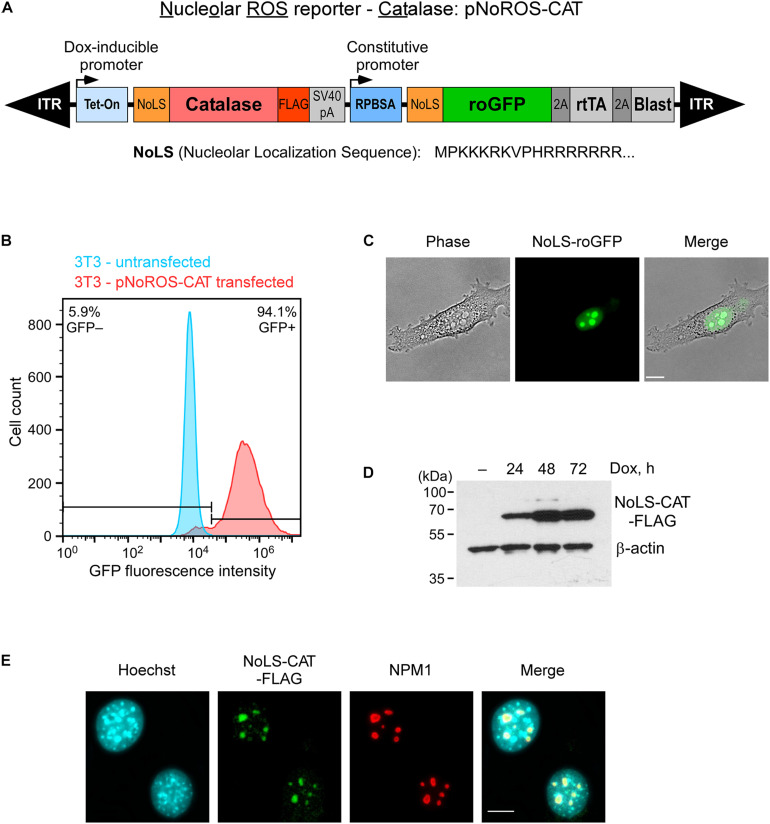
Targeting of roGFP and catalase to the nucleolus. **(A)** Schematic of the genome-integrated portion of pNoROS-CAT flanked by inverted terminal repeats (ITR). The Dox-inducible Tet-On promoter controls expression of a mouse catalase gene fused with a nucleolar localization sequence (NoLS) at the N terminus and a FLAG tag at the C terminus of the protein. The constitutive RPBSA promotor drives expression of a polycistronic sequence encoding NoLS-roGFP, tetracycline transactivator gene (rtTA), and blasticidin resistance marker, all separated by 2A self-cleaving peptide elements. **(B)** Flow cytometric analysis of a pool of 3T3 cells transfected with pNoROS-CAT following a single round of blasticidin selection. **(C)** Phase-contrast and fluorescent live imaging of cells from a pNoROS-CAT-transfected cell pool shows NoLS-roGFP accumulation in the nucleolus. **(D)** Western blot of NoLS-CAT induction in cells stably transfected with pNoROS-CAT. NoLS-CAT was detected with FLAG antibody, endogenous β-actin is a loading control. **(E)** Immunofluorescent analysis shows NoLS-CAT targeting to the nucleolus. NoLS-CAT was detected using FLAG antibody, the nucleolus was visualized with antibody against endogenous NPM1. Nuclei were counterstained with Hoechst 33342. In **(C,E)**, representative microscopic fields are shown. Scale bars, 10 μm.

To begin to characterize the effects of redox perturbations on pre-rRNA synthesis and maturation, we constructed a vector, termed pNoROS, that incorporates two expression cassettes, both of which carry an efficient nucleolar localization sequence (NoLS) derived from a previous study by Martin and colleagues (Martin et al., 2015). As illustrated in Figure 1A, a constitutive promoter in pNoROS directs expression of the redox-sensitive roGFP, which exhibits different excitation maxima for its reduced and oxidized forms (Dooley et al., 2004), thus providing a readout of the redox environment specific to the nucleolus. The second, tightly regulated Tet-On promoter is used for the doxycycline (Dox)-inducible expression of a protein that provides a means to regulate the redox environment in the nucleolus. To modulate H_2_O_2_ levels, we placed a FLAG-tagged coding sequence of catalase, a highly efficient H_2_O_2_ scavenger, into the Dox-regulated cassette, thus generating pNoROS-CAT (Figure 1A). To achieve stable expression in mammalian cells, pNoROS utilizes an enhanced Sleeping Beauty transposon system (Kowarz et al., 2015). When supplied with the Sleeping Beauty transposase, the flanking internal terminal repeats (ITR) in pNoROS constructs direct the efficient integration of the expression cassettes into genomic DNA.

To validate expression and nucleolar targeting of the roGFP and catalase, we cotransfected pNoROS-CAT into mouse 3T3 cells together with the non-integrating plasmid pCMV(CAT)T7-SB100 (Mátés et al., 2009) that provides the Sleeping Beauty transposase, followed by blasticidin selection for cells harboring the stably integrated pNoROS-CAT. Based on a flow cytometry analysis of the transfected cell population, >90% blasticidin-selected cells exhibited roGFP expression (Figure 1B). A microscopical examination of these blasticidin-resistant cells showed that the roGFP signal was concentrated in the nucleolus (Figure 1C). Western blot analysis of FLAG-tagged NoLS-CAT in cell extracts confirmed that it can be tightly regulated through Dox induction (Figure 1D). Immunofluorescence analysis of the transfected cells verified the nucleolar accumulation of NoLS-CAT (Figure 1E).

### H_2_O_2_ Alters the Redox State of the Nucleolus

**FIGURE 2:**
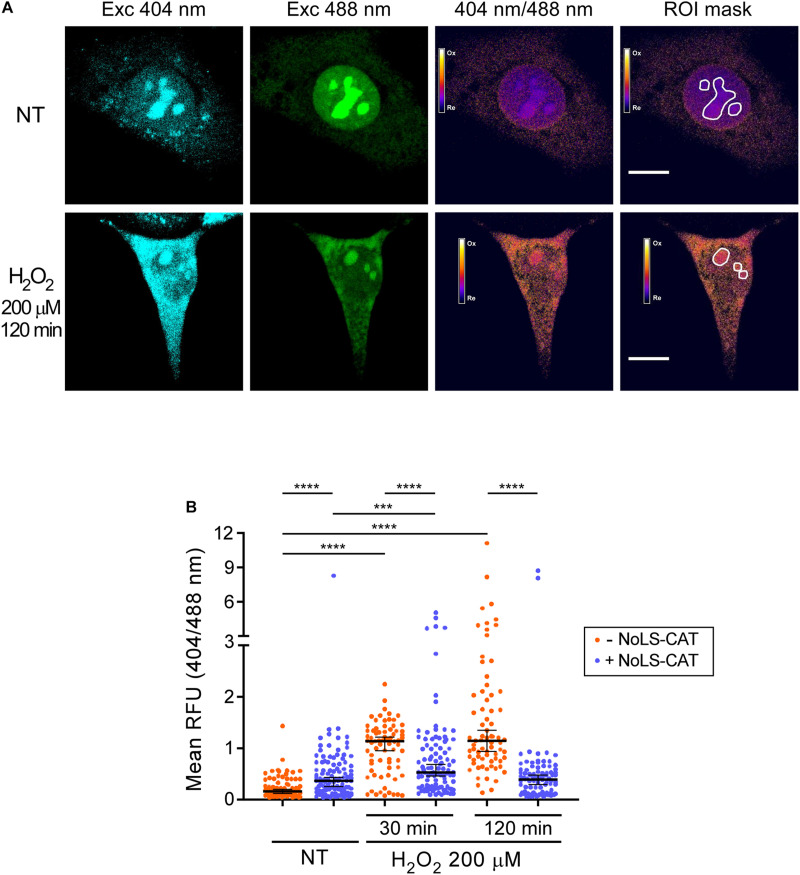
H_2_O_2_ promotes oxidation of the nucleolus. **(A)** Example of the ratiometric analysis of the nucleolar redox state. Live images of cells expressing NoLS-roGFP were taken before and after a 2 h treatment with 200 μM H_2_O_2_. Emission at 515 nm was measured using the indicated excitation wavelengths to calculate the 404/488 nm excitation ratio of NoLS-roGFP. The white lines demarcate the Regions of Interest (ROIs) used to measure NoLS-roGFP signal in the nucleolus. Scale bars, 10 μm. For an example of a wider microscopic field of the cells, see Supplementary Figure 2. **(B)** Induction of NoLS-CAT counteracts the oxidation of the nucleolus induced with H_2_O_2_. The 404/488 nm excitation ratio of the nucleolar roGFP was measured in pNoROS-CAT-transfected pools grown either without Dox (–NoLS-CAT) or in the presence of 1 μg/ml Dox for 24 h (+NoLS-CAT) prior to the indicated H_2_O_2_ treatments. For each set of conditions, 70–110 nucleoli from a total of 30–40 cells were analyzed. The horizontal line represents the median, error bars show 95% CI. Statistical significance was assessed by pairwise Mann-Whitney tests. ****P < 0.0001, ***P < 0.001 (two-tailed values).

The redox-sensitive roGFP used in pNoROS-CAT incorporates two built-in cysteine residues that can form a disulfide bridge when oxidized, deprotonating the inner chromophore. This increases the emission intensity of the protein when excited at wavelengths near 400 nm while decreasing the emission when excited at around 490 nm (Dooley et al., 2004). As a model of oxidative stress, we exposed NIH 3T3 cells transfected with pNoROS-CAT to 200 μM H_2_O_2_. The mild concentration of H_2_O_2_ was chosen to avoid cell detachment, which would be incompatible with live-cell microscopy, and was also below the dose that induces death in these cells. We next performed live-cell imaging to assess the redox state of the intranucleolar environment by measuring the differential emission of NoLS-roGFP in the nucleolus at 515 nm when excited using two laser sources, 404 nm and 488 nm (Figure 2A and Supplementary Figure 1).

The ratio of the roGFP emission when excited at 404 vs 488 nm serves as an indicator of the redox state of the protein’s local environment (Dooley et al., 2004). There is substantial variability in this ratio for NoLS-GFP at the level of individual cells (Supplementary Figure 2). However, when multiple cells in each transfected pool were analyzed, we observed statistically robust differences depending on whether the cells were subjected to an H_2_O_2_ challenge and whether NoLS-CAT was induced (Figure 2B). In cells that did not express NoLS-CAT, 200 μM H_2_O_2_ caused a significant increase in the 404/488 nm excitation ratio of NoLS-roGFP fluorescence when tested 30–120 min after the treatment, indicating elevated oxidation of the intranucleolar space. Induction of NoLS-CAT, a scavenger for H_2_O_2_, strongly suppressed the increase in the NoLS-roGFP 404/488 nm ratio (Figure 2B). Interestingly, expression of catalase alone resulted in a small but reproducible increase in the oxidation of the nucleolus. It was previously reported that cells engineered to express catalase were sensitive to bleomycin, doxorubicin and paraquat (Speranza et al., 1993), which was attributed primarily to the catalase-driven generation of dioxygen (O_2_) in these cells. Thus, we speculate that NoLS-CAT activity might similarly provoke an increase in the local O_2_ concentration leading to an elevated basal oxidation state of the nucleolus. Although this property might limit catalase useability as a broad-purpose antioxidant agent, the robust mitigation of the H_2_O_2_ spike during experimental treatments (Figure 2B) shows that NoLS-CAT can be a useful tool to investigate acute effects of H_2_O_2_ on nucleolar processes.

### Exposure to H_2_O_2_ Leads to Increased 8-oxo-G Levels in Nucleolar RNA

**FIGURE 3:**
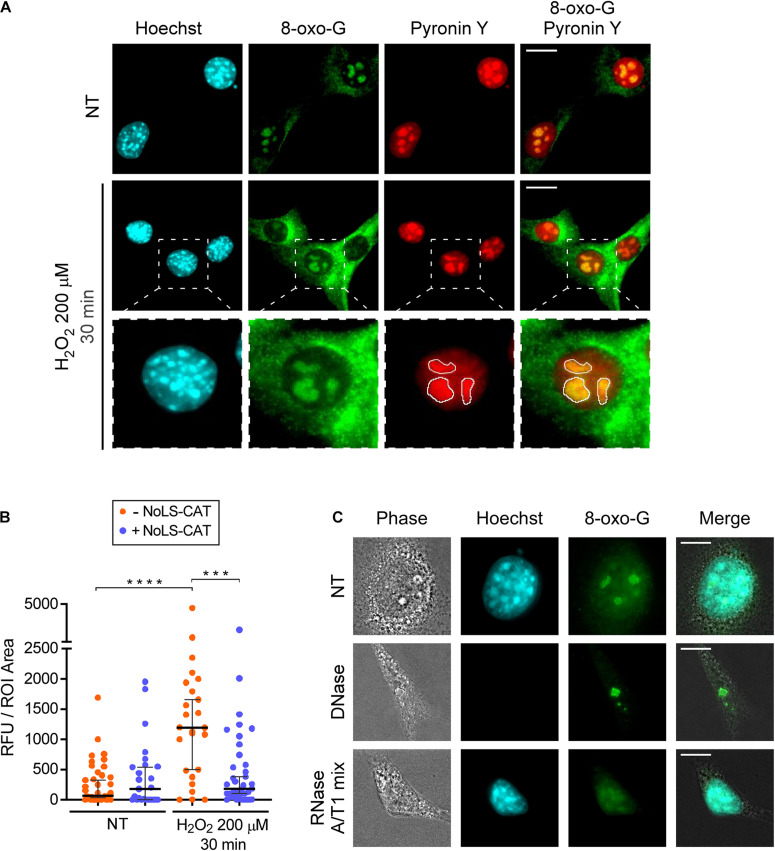
H_2_O_2_ increases 8-oxo-G in nucleolar RNA. **(A)** Microscopic analysis of DNA (Hoechst 33342 signal), RNA (pyronin Y signal) and 8-oxo-G (immunostaining with the monoclonal 15A3 antibody). Representative images are shown for cells not treated with H_2_O_2_ (basal oxidation levels) and cells treated with 200 μM H_2_O_2_ for 30 min. The white lines in the bottom row demarcate nucleolar ROIs based on the pyronin Y signal. For an example of a wider field, see Supplementary Figure 3. **(B)** NoLS-CAT confers protection from an increase in nucleolar 8-oxo-G caused by H_2_O_2_ exposure. *n* = 39, 25, 25, 27 individual nucleoli analyzed (from left to right), the horizontal center line represents the median, error bars indicate 95% CI. Mann-Whitney test was used for pairwise comparisons. ****P < 0.0001, ***P < 0.001 (two-tailed values). **(C)** Representative images of cells subjected to DNase or RNase treatments (NT, non-treated control). Note that for the enzymatic treatments, cells are permeabilized with detergents prior to their fixation, accounting for the cells’ collapsed appearance in the resulting images. After fixation, cells were stained with anti-8-oxo-G antibody. Hoechst staining visualizes DNA in the nucleus. For an example of a wider field, see Supplementary Figure 4. Scale bars, 10 μm.

Upon exposure to oxidants, numerous types of chemical modifications are known to occur in nucleic acids (Küpfer and Leumann, 2014). 8-Oxo-7,8-dihydroguanosine (8-oxo-G) is one abundant lesion type (Radak and Boldogh, 2010) that can be detected in both DNA and RNA using previously developed monoclonal antibodies (Park et al., 1992). We wondered whether 8-oxo-G would be present in the nucleolus after an H_2_O_2_ challenge, which, as shown above, leads to nucleolar oxidation. To test this, we performed immunofluorescence staining of pNoROS-CAT-transfected cell pools using the well-characterized 15A3 monoclonal antibody raised against 8-oxo-G (Park et al., 1992). In addition to immunostaining, samples were stained with the DNA-specific dye Hoechst 33342 followed by a brief incubation with pyronin Y, a dye that stains both DNA and RNA. Because staining with Hoechst reduces pyronin binding to DNA, this technique allows visualization of RNA-rich areas, such as the nucleolus (Darzynkiewicz et al., 1987). We selected nucleolar ROIs based on the pyronin Y signal (Figure 3A) and performed quantification of 8-oxo-G staining intensity in the nucleoli of multiple cells. As shown in Figure 3B, a 30-min H_2_O_2_ treatment significantly increased the mean intensity of the nucleolar 8-oxo-G signal.

Given that the nucleolus contains more RNA than DNA, we reasoned that the nucleolar 8-oxo-G signal we observed (Figure 3A) could be due to oxidative modifications in RNA. To determine which type of nucleic acid was responsible for the 8-oxo-G antibody reactivity with the nucleolus, we permeabilized cells prior to their fixation, and carried out digestion with either DNase or RNase. Based on a control staining with the Hoechst dye, treatment with DNase, but not RNase, led to a loss of nuclear DNA (Figure 3C). In contrast, the nucleolar signal of the antibody survived the DNase treatment, whereas the RNase treatment completely destroyed it (Figure 3C and Supplementary Figure 4). Because rRNA contains numerous chemical modifications (Taoka et al., 2018), we further wanted to exclude the possibility that the antibody binding to nucleolar RNA could be an artifact of a previously uncharacterized cross-reactivity toward some RNA modification unconnected with ROS effects. To this end, we used the conditional expression of NoLS-CAT to lower H_2_O_2_ concentration inside the nucleolus. As shown in Figure 3B, the increase in nucleolar staining after H_2_O_2_ treatment was completely prevented by NoLS-CAT expression. Thus, the 15A3 antibody’s reactivity toward nucleolar RNA correlates with the oxidative state of the nucleolus, leading us to conclude that even a mild H_2_O_2_ stress is capable of significantly increasing oxidative damage in nucleolar RNA.

### Distinct Steps in Pre-rRNA Maturation Are Affected by H_2_O_2_ Exposure

**FIGURE 4:**
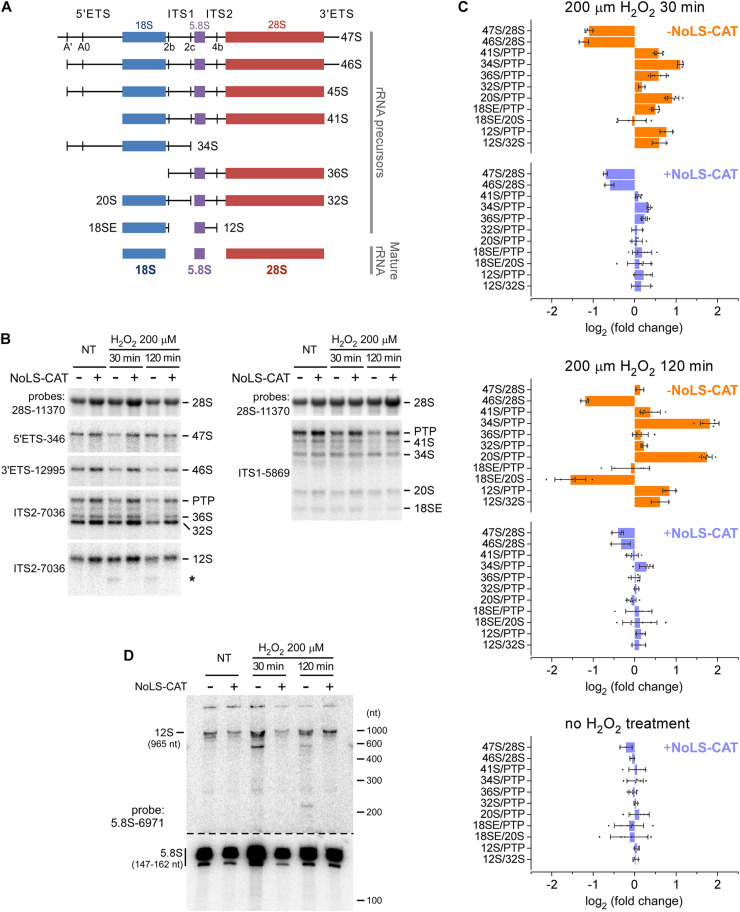
Alterations in pre-rRNA processing after H_2_O_2_ exposure. **(A)** The main pre-rRNA processing intermediates in mouse cells. Major cleavage sites in the primary 47S pre-rRNA are shown at the top. **(B)** Northern blot analysis of pre-rRNA processing. pNoROS-CAT-transfected pools were either not treated (NT) or treated with H_2_O_2_ for the indicated times. For NoLS-CAT induction, Dox was added to medium 24 h prior to H_2_O_2_ treatment. RNA was resolved on 1% agarose-formaldehyde gels, blotted onto nylon membranes, and hybridized with radiolabeled probes. Representative hybridizations are shown as examples. PTP refers to the band consisting of the *p*rimary *t*ranscript (47S) plus its derivatives 46S and 45S, all three of which comigrate on agarose gels. Asterisk marks an aberrant product of incomplete 3′ processing of 12S pre-rRNA appearing after H_2_O_2_ treatment. **(C)** RAMP plots showing changes in precursor ratios relative to those in control cells that were neither exposed to H_2_O_2_ nor expressed NoLS-CAT. Individual data points correspond to values obtained from three biological replicates; some hybridizations were further performed in two technical repeats. Bars show mean values, error bars show sd. **(D)** Northern blot analysis of the 12S to 5.8S processing. RNA was resolved on an 8% polyacrylamide-urea gel, transferred onto a membrane, and hybridized with a 5.8S rRNA probe. To avoid signal saturation by the abundant 5.8S rRNA, the bottom part of the membrane was cut out and exposed separately for a ∼10-fold shorter time than the top part containing 5.8S precursors.

In mammalian cells, 28S and 5.8S rRNA in the 60S ribosomal subunit and 18S rRNAs in the 40S subunit derive from a single 47S pre-rRNA, processed to the mature rRNAs through a complex sequence of posttranscriptional events (Henras et al., 2015; Baßler and Hurt, 2019). To investigate whether exposure to H_2_O_2_ affects the pre-rRNA maturation process, we examined steady-state levels of major processing intermediates by using oligonucleotide probes that hybridize to specific sites within the pre-rRNA transcript (Figures 4A,B and Supplementary Table 1). RNA for this analysis was extracted from cell pools harboring pNoROS-CAT that were either induced with Dox for 24 h to express NoLS-CAT or left uninduced and treated or not with 200 μM H_2_O_2_ for 30 min to 2 h. To accurately assess the state of pre-rRNA processing, we analyzed hybridization data using the previously described quantification workflow termed the ratio analysis of multiple precursors (RAMP) (Wang et al., 2014).

The resulting RAMP profiles (Figure 4C) revealed a complex picture of changes in pre-rRNA maturation occurring after an H_2_O_2_ challenge. When analyzed at 30 min (Figure 4C, top), there was a marked drop in the 47S primary transcript and its earliest processing product, 46S pre-rRNA (see Figure 4A), consistent with a lowered transcription of pre-rRNA. The 47S levels returned to baseline after 120 min, but 46S remained low (Figure 4C, middle), likely because 47S processing to 46S, which normally occurs through a rapid cleavage of the 5′ end of the transcript at site A′ (Figure 4A), remained inhibited.

To evaluate downstream processing events, we examined ratios of different processing intermediates relative to the reference level of the “primary transcript plus” (PTP), the combination of the early 47S–45S pre-rRNAs that comigrate on gels (Wang et al., 2014). At 30 min after the H_2_O_2_ treatment, we found a significant increase in the 34S/PTP and 20S/PTP ratios, which further increased at 120 min (Figure 4C, top and middle). Both 34S and 20S are precursors for 18S rRNA, and their accumulation points to the anomalous dynamics of 40S subunit assembly. In contrast to these 18S rRNA precursors, there was little change in the 32S pre-rRNA, a precursor to 5.8S and 28S rRNAs. However, H_2_O_2_ exposure appeared to strongly affect 60S assembly steps past 32S formation, during which 5.8S rRNA is generated from its precursor 12S pre-rRNA. First, 12S accumulated in H_2_O_2_-treated cells, as judged from the increases of the 12S/PTP and 12S/32S ratios (Figure 4C, top and middle). Second, a novel RNA species was detectable below the 12S pre-rRNA band in hybridization assays utilizing an ITS2 probe (Figure 4B, asterisk). To better characterize this aberrant intermediate, we separated RNA on a polyacrylamide gel and hybridized the blot with a 5.8S rRNA-specific probe (Figure 4D). This revealed accumulation of an RNA ∼600 nt in length as well as a smear of shorter lower-intensity products following the H_2_O_2_ challenge. In cells, 12S is processed to 5.8S rRNA through a series of 3′ exonucleolytic trimming reactions (Lubas et al., 2011; Schillewaert et al., 2012; Thoms et al., 2015; Pirouz et al., 2019). Our data indicate that H_2_O_2_ stress causes the impairment of this processing step.

When cells were induced to express NoLS-CAT before H_2_O_2_ treatment, the shifts in precursor levels described above were substantially attenuated and in many cases were not significantly different from the baseline (Figure 4C, top and middle, compare profiles for + NoLS-CAT cells). This corroborates the conclusion that exposure to H_2_O_2_ at doses that induce nucleolar oxidation (Figure 2) and accumulation of 8-oxo-G in nucleolar RNA (Figure 3) also disrupts normal processing of pre-rRNA. Notably, induction of NoLS-CAT by itself did not significantly perturb pre-rRNA processing (Figure 4C, bottom).

## Discussion

In this work, we developed novel tools to investigate oxidative stress-driven effects on pre-rRNA metabolism in mammalian cells. The pNoROS-based constructs, which target the redox-sensitive roGFP to the nucleolus, should facilitate studying the redox state of this metabolically active nuclear compartment under diverse types of challenges. Our current results also demonstrate that unmitigated H_2_O_2_ reaching the nucleolus has functional consequences, as evidenced by increased levels of the oxidation marker 8-oxo-G in nucleolar RNA and impaired pre-rRNA processing.

Subjecting mouse 3T3 cells to a brief pulse of H_2_O_2_ resulted in a changed ratio between the oxidized and reduced forms of the nucleolar redox reporter NoLS-roGFP, attesting to the oxidation of the intranucleolar space (Figure 2). H_2_O_2_ is naturally generated in cells in a wide range of metabolic processes and can also enter cells by crossing plasma membranes from the extracellular environment (Imlay, 2008; Sies, 2014). While H_2_O_2_ is relatively unreactive with biomolecules, it can give rise to the highly reactive hydroxyl radical through Fenton chemistry, with iron ions likely contributing the most to the destructive potential of H_2_O_2_ in biological systems (Mahaseth and Kuzminov, 2017). It is believed that by this mechanism, exposure to H_2_O_2_ produces a variety of chemical modifications in nucleic acids, with previous studies largely focusing on DNA as the principal target (Aruoma et al., 1989). Using immunofluorescent analysis with antibodies reactive with nucleic acids containing 8-oxo-G, we observed prominent staining of the nucleolus in 3T3 cells, further increased when the cells were exposed to H_2_O_2_. Because the nucleolus is primarily the site of ribosome biogenesis and has little DNA compared with the nucleoplasm, this result suggested that nucleolar RNA could be the major target of the H_2_O_2_-driven redox disequilibrium. Consistent with this idea, we found that nucleolar 8-oxo-G staining was sensitive to RNase digestion, but not to DNase (Figure 3B). We do not know yet how much of the affected RNA in the nucleolus is rRNA. While the nucleolus serves as the hub of ribosome synthesis, other classes of RNA are known to be present in this organelle (Kaliatsi et al., 2020). It will be of great interest to comprehensively characterize different nucleolar RNAs with respect to oxidative stress-induced modifications.

Perturbations of ribosome biogenesis in cells exposed to H_2_O_2_ can be inferred based on altered steady-state ratios of several different pre-rRNA processing intermediates. Our profiling of the pre-rRNA ratios (Figure 4) suggests that both pre-rRNA transcription and posttranscriptional processing are affected in 3T3 cells subjected to H_2_O_2_ stress. It is also evident from this analysis, however, that H_2_O_2_ exposure does not globally shut down pre-rRNA maturation, but rather inhibits specific steps in this pathway. In particular, the maturation of pre-5.8S rRNA appears to be highly sensitive to H_2_O_2_. The 3′ end of 5.8S rRNA in the eukaryotic 60S ribosomal subunit is formed via progressive 3′–5′ exoribonucleolytic trimming performed by the RNA exosome complex that docks to the nascent pre-60S subunit (Thoms et al., 2015; Schuller et al., 2018). Deficiencies in various exosome components were previously observed to produce a ladder of 3′-extended 5.8S precursors (Schillewaert et al., 2012). The major pre-5.8S rRNA form accumulating in murine cells after depletion of exosome subunits was reported to have a 106-109-nt 3′-end extension (Pirouz et al., 2019), which corresponds to an RNA of 250–270 nt in total length. Because the largest aberrant 5.8S precursor observed in H_2_O_2_-treated cells is close to 600 nt (Figure 4D), it appears unlikely that the inhibition of exosome activity *per se* can explain this processing defect. Another possibility is that abnormal remodeling of the pre-60S ribosomal subunit may result in a failure to recruit the exosome, its cofactors (Lubas et al., 2011), or cause steric hindrance to exosome progression. Thus, one goal for the future studies would be to identify precisely which components in the pre-rRNA processing machinery are vulnerable to H_2_O_2_ stress.

A wide range of chemical inhibitors, including many clinically important anticancer drugs, cause impairment of pre-rRNA transcription and processing, often associated with a physical disruption of the nucleolus (Burger et al., 2010; Awad et al., 2019). Unlike those previously studied inhibitors, inducers of oxidative stress are currently not well understood with respect to their impact on pre-rRNA metabolism and nucleolus integrity. While our results show that certain steps in pre-rRNA processing in mouse 3T3 cells are highly H_2_O_2_-sensitive, the nucleolus as a whole appears to tolerate the H_2_O_2_ challenge fairly well. Because efficient translation of stress-response proteins could conceivably depend on the synthesis of new ribosomes, the resilience of the nucleolus could be important for the cell’s overall ability to withstand oxidative stress. At the same time, the finding that supplying catalase to the nucleolus ameliorates pre-rRNA processing defects caused by H_2_O_2_ (Figure 4) demonstrates intrinsic limitations to the nucleolar built-in antioxidant defenses.

In this study, we focused our investigation on H_2_O_2_. How other ROS generated in cells affect pre-rRNA metabolism remains to be elucidated. One biologically important ROS type in both homeostatic and stress situations is superoxide (Imlay, 2008), detoxified in part by superoxide dismutases (SODs). We have recently found that a fraction of mouse SOD1 localizes to the nucleolus and facilitates the generation of 60S ribosomal subunits in lung cancer cells (Wang et al., 2021). Thus, it appears that protecting pre-rRNA and the ribosome-building pathway from oxidative damage requires synergistic activities of different types of antioxidant mechanisms.

## Data Availability Statement

The raw data supporting the conclusions of this article will be made available by the authors, without undue reservation.

## Author Contributions

RS and DP conceived and designed experiments in this study, analyzed the data and prepared figures, and edited the manuscript. RS performed most experiments. CB developed the 8-oxo-G antibody staining protocol. DP wrote the manuscript. All authors contributed to the article and approved the submitted version.

## Conflict of Interest

The authors declare that the research was conducted in the absence of any commercial or financial relationships that could be construed as a potential conflict of interest.
