# Supplementary material for: Effects of Hydrogen Peroxide Stress on the Nucleolar Redox Environment and Pre-rRNA Maturation
Source: Front Mol Biosci. 2021 Apr 26;8:678488. doi: 10.3389/fmolb.2021.678488 (PMC8107432; doi:10.3389/fmolb.2021.678488)
Supplement: Supplementary file 1 [file Image_1.PDF]

Supplementary Material

Effects of hydrogen peroxide stress on the nucleolar redox environment and pre-rRNA maturation

Russell T. Sapio, Chelsea J. Burns, and Dimitri G. Pestov

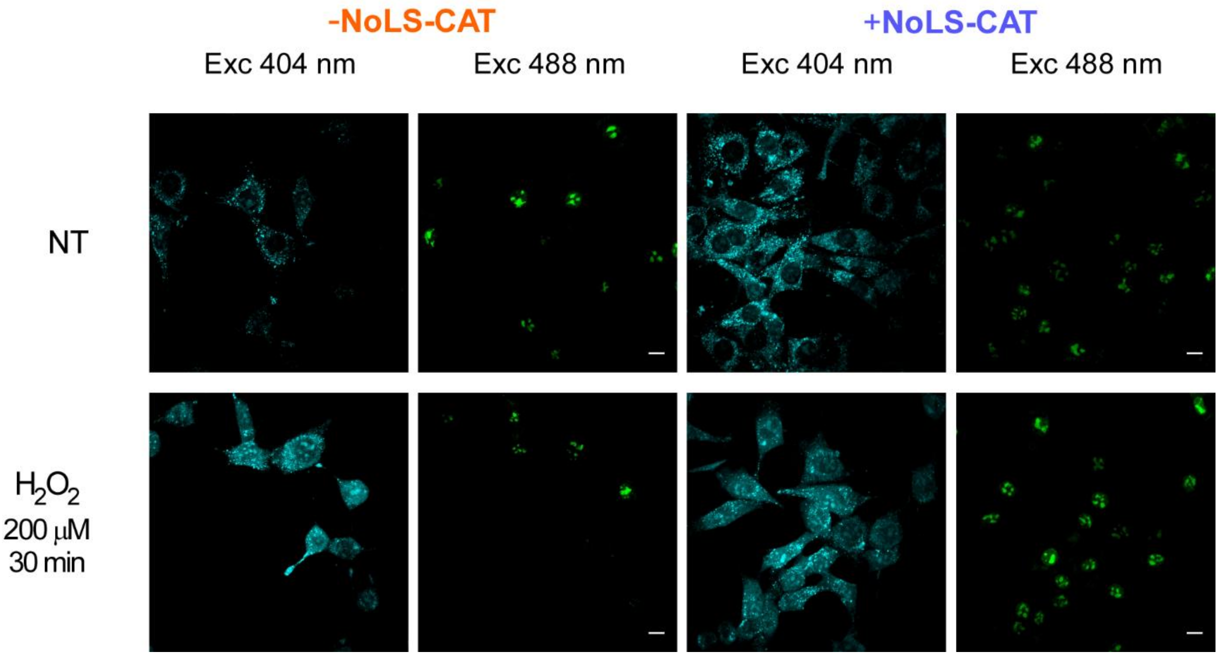

Supplementary Figure S1. Wide field views of pNoROS-CAT-transfected cell pools. For each field, live-cell confocal images were acquired at 515 nm with excitation at 404 nm and 488 nm. For the induction of NoLS-CAT, Dox was added to cell culture medium 24 h prior to H_2O_2 treatment. Scale bars, 10 µm.

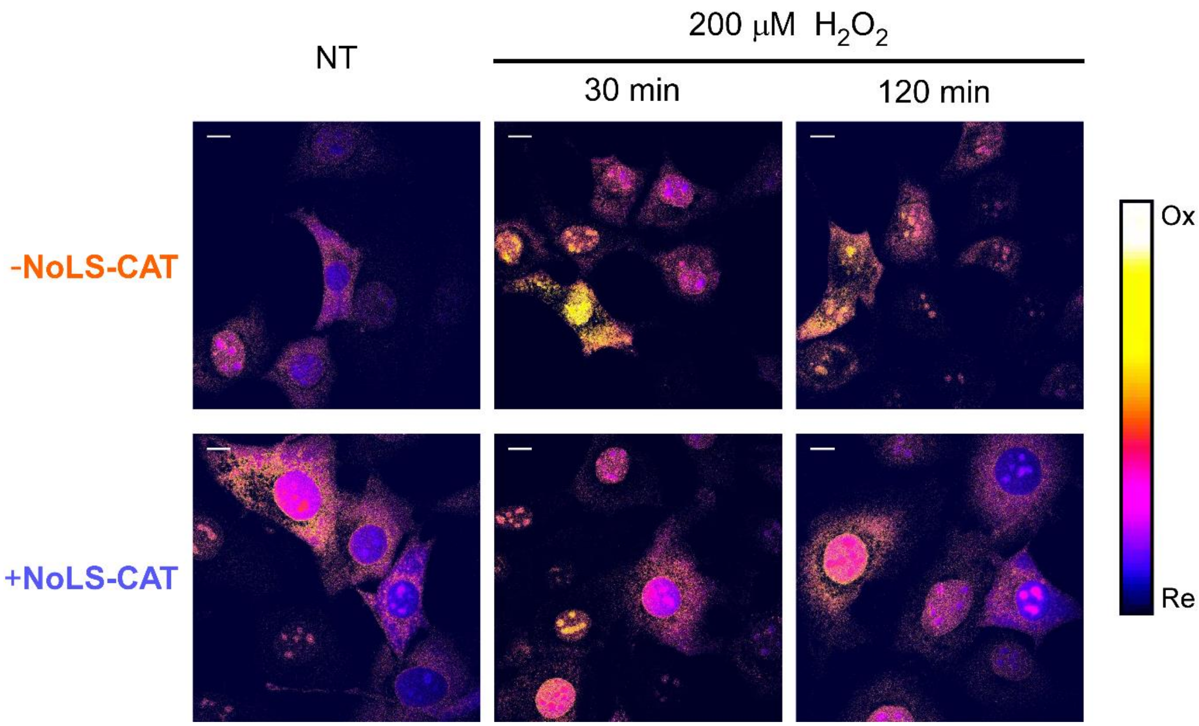

Supplementary Figure S2. An example of the 404/488 nm ratiometric analysis in a population of 3T3 cells transfected with pNoROS-CAT. Emission was measured at 515 nm with excitation at 404 nm and 488 nm. The calculated 404/488 nm ratio is presented as a heatmap. For the induction of NoLS-CAT, Dox was added to cell culture medium 24 h prior to H_2O_2 treatment. Scale bars, 10 µm.

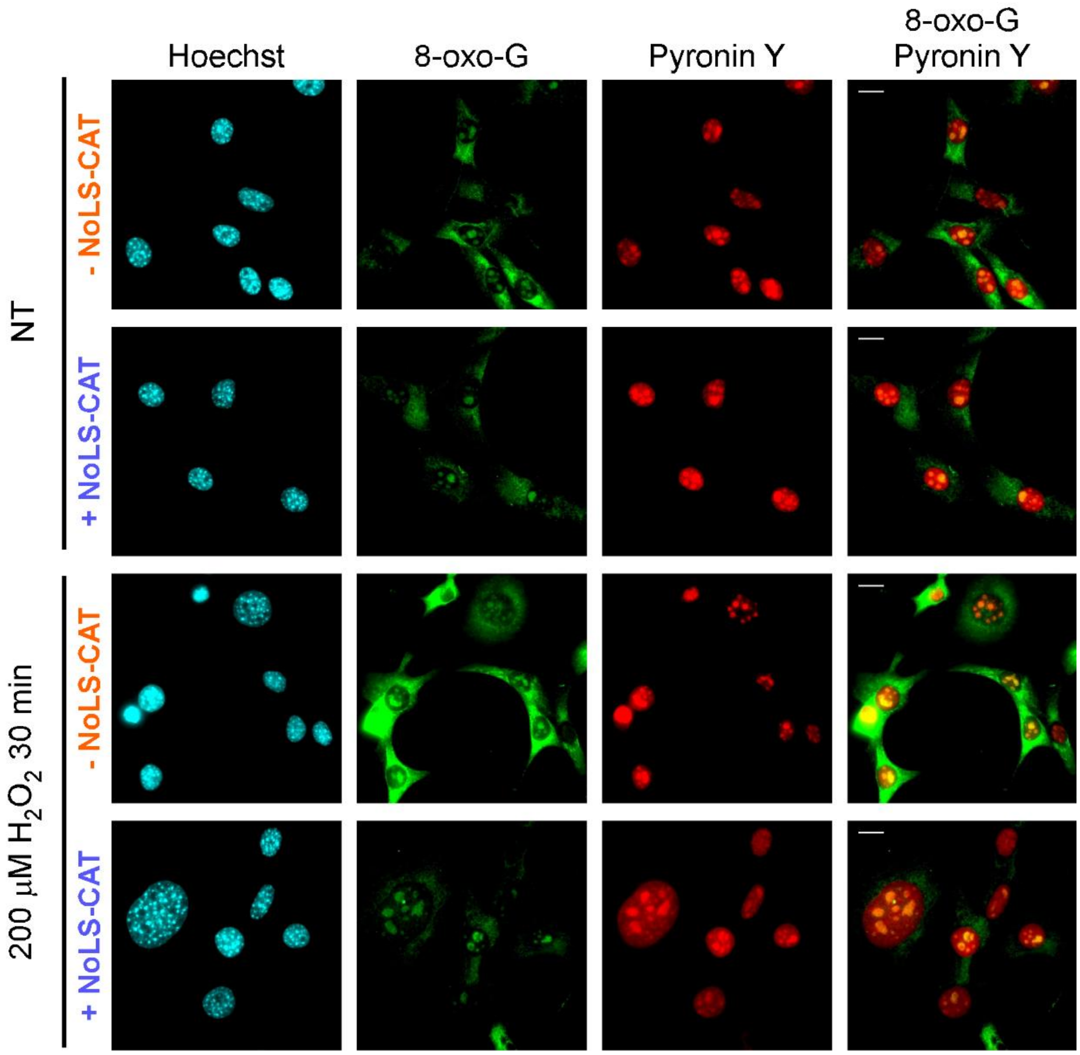

Supplementary Figure S3. Immunostaining of pNoROS-CAT-transfected cell pools with 8-oxo-G antibody, in combination with Hoechst 33342/pyronin Y staining. For NoLS-CAT induction, Dox was added to cells 24 h prior to their treatment with 200 µM H_2O_2. These images are representative of the data presented in Figure 3A-B. Scale bars, 10 µm.

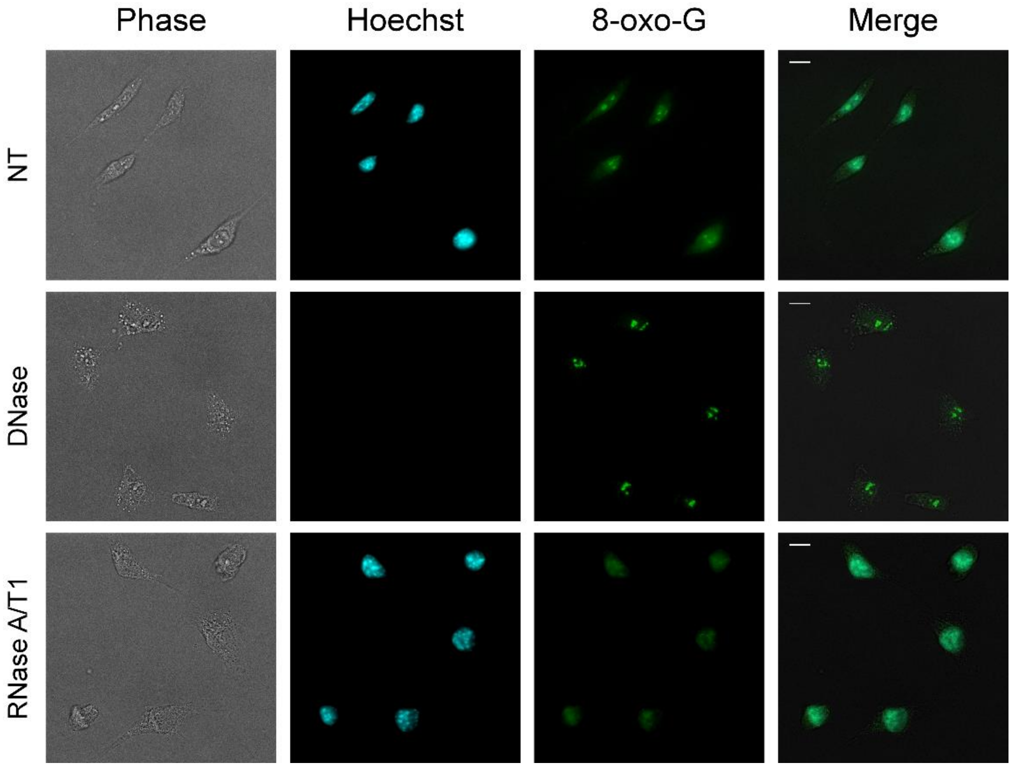

Supplementary Figure S4. Wide fields of cells subjected to the DNase and RNase treatments. Cells were stained with anti-8-oxo-G antibody 15A3. Nuclear DNA was visualized with Hoechst 33342. NT, no enzymatic treatment.
