# Supplementary material for: Effects of Hydrogen Peroxide Stress on the Nucleolar Redox Environment and Pre-rRNA Maturation
Source: Front Mol Biosci. 2021 Apr 26;8:678488. doi: 10.3389/fmolb.2021.678488 (PMC8107432; doi:10.3389/fmolb.2021.678488)
Supplement: Supplementary file 2 [file Table_1.DOCX]

**Supplementary Table 1. Oligonucleotide probes used in this study.**

| Probe | Sequence (5'-3') | Position in the reference mouse rDNA sequence BK000964, nt |
| --- | --- | --- |
| 5'ETS-346 | agagaaaagagcggaggttcgggactccaa | 346-375 |
| ITS1-5869 | tcctccacagtctcccgtttaatgatc | 5869-5895 |
| 5.8S-6971 | gcaagtgcgttcgaagtgt | 6971-6989 |
| ITS2-7036 | cgatcaatcgcgtcacccgctgcggtgggt | 7036-7065 |
| 28S-11370 | ttctaagtcggctgctaggc | 11370-11389 |
| 3'ETS-12995 | agagcgacggaaggggaaagagaaacgaac | 12995-11389 |
